# Metabolome-Driven Regulation of Adenovirus-Induced Cell Death

**DOI:** 10.3390/ijms22010464

**Published:** 2021-01-05

**Authors:** Anastasia Laevskaya, Anton Borovjagin, Peter S. Timashev, Maciej S. Lesniak, Ilya Ulasov

**Affiliations:** 1 Group of Experimental Biotherapy and Diagnostic, Institute for Regenerative Medicine, World-Class Research Center “Digital Biodesign and Personalized Healthcare”, Sechenov First Moscow State Medical University, 119991 Moscow, Russia; ayaksveal@yandex.ru; 2 Department of Biomedical Engineering, University of Alabama at Birmingham, Birmingham, AL 35294, USA; aborovjagin@gmail.com; 3 Institute for Regenerative Medicine, World-Class Research Center “Digital Biodesign and Personalized Healthcare”, Sechenov First Moscow State Medical University, 119991 Moscow, Russia; timashev_p_s@staff.sechenov.ru; 4 Department of Polymers and Composites, N.N.Semenov Institute of Chemical Physics, 4 Kosygin St., 119991 Moscow, Russia; 5 Chemistry Department, Lomonosov Moscow State University, Leninskiye Gory 1-3, 119991 Moscow, Russia; 6 Department of Neurological Surgery, Northwestern University, Chicago, IL 60601, USA; maciej.lesniak@northwestern.edu

**Keywords:** autophagy, viruses, metabolites

## Abstract

A viral infection that involves virus invasion, protein synthesis, and virion assembly is typically accompanied by sharp fluctuations in the intracellular levels of metabolites. Under certain conditions, dramatic metabolic shifts can result in various types of cell death. Here, we review different types of adenovirus-induced cell death associated with changes in metabolic profiles of the infected cells. As evidenced by experimental data, in most cases changes in the metabolome precede cell death rather than represent its consequence. In our previous study, the induction of autophagic cell death was observed following adenovirus-mediated lactate production, acetyl-CoA accumulation, and ATP release, while apoptosis was demonstrated to be modulated by alterations in acetate and asparagine metabolism. On the other hand, adenovirus-induced ROS production and ATP depletion were demonstrated to play a significant role in the process of necrotic cell death. Interestingly, the accumulation of ceramide compounds was found to contribute to the induction of all the three types of cell death mentioned above. Eventually, the characterization of metabolite analysis could help in uncovering the molecular mechanism of adenovirus-mediated cell death induction and contribute to the development of efficacious oncolytic adenoviral vectors.

## 1. Introduction

Metabolomics is a field of science that studies the entire set of low molecular weight organic compounds or small molecules, also called metabolites, in individual cells or biological samples and changes thereof by using chemical assays and data analyses, which commonly includes nuclear magnetic resonance spectroscopy and different types of mass spectrometry [1]. In contrast to genomics and proteomics, metabolomics analyzes the end-products or outcomes of biochemical processes that occur in the whole cell, as opposed to such metabolic processes themselves. The subject of a global metabolite analysis (Box 1) in a particular cell is the cell’s metabolome, which essentially determines the cell’s phenotype. For instance, the change in the cell’s metabolome associated with the development of breast cancer correlates with the alteration in the cancer cell’s shape [2]. Metabolome measurements provide a complete picture of all physiological processes occurring in the cell within a specific time period. Alterations in the metabolic profiles can be induced by multiple factors that include: environmental, such as nutrition supply; genetic, such as mutations; and microbiological, such as viral infection, which is the most important for the subject of this review.

Viral infection greatly affects cell’s metabolome because viruses “usurp” the host (infected) cell’s metabolism for their life (replication) cycle. The interaction of virus-specific proteins with those of the host cell creates an environment that is essential for viral replication and is impossible without metabolomic shifts. For instance, replication of oncolytic adenovirus OBP-301 triggers the production of uric acid in infected cells, which induces immune responses that promote immunogenic cell death [3]. Another example of a virus-induced metabolic response is an infection of microglial cells with Zika virus that is accompanied by an increase in the levels of lysophosphatidylcholine, which in turn triggers apoptosis [4]. Thus, a viral life cycle in the host cell can lead to various types of cell death that are associated with alterations in the cell’s metabolomic profile. It is not yet fully understood whether specific metabolites (Box 2) induce a particular type of cell death, or conversely, a specific type of programmed cell death predetermines particular metabolomic shifts. Furthermore, uncovering which metabolites are involved in the induction of a particular type of cell death could aid in defining new targets for oncolytic virotherapy applications.

A wide variety of viruses from different families, including human adenovirus (Ad), have been used for oncolytic virotherapy applications. The adenovirus represents a promising therapeutic to treat cancers in combination with chemo/chemoradiation therapy or surgical resection owing to their high-load capacity as delivery vectors and high infectivity [5]. Constructed by means of genetic engineering, conditionally replicating adenoviruses (CRAds) replicate selectively and exhibit oncolytic activity only in cancer cells while sparing normal cells, owing to their engineered transductional and/or transcriptional targeting properties. Such replication-competent Ad vectors induce oncolysis via various mechanisms including autophagy, apoptosis, necrosis, pyroptosis and an immune response. As previously stated, all types of cell death are associated with metabolomic alterations. Implementing metabolite analysis in the assessment of the correlation between cellular metabolites and the adenovirus-mediated oncolysis has the potential to uncover new targets for adenovirus-based virotherapy and improve its efficacy. However, it is important to consider that metabolic shifts largely depend on the type of cell line and the infecting viral vector. For this reason, one should refrain from any generalizations with regard to the metabolome of adenovirus-infected cells while reading this review.

## 2. Metabolites Associated with the Adenovirus-Induced Autophagic Cell Death

Autophagy is a programmed cytoprotective mechanism for degrading unnecessary or dysfunctional intracellular components in a unique cytoplasmic organelle, known as an autophagosome. The autophagy mechanism is triggered when the cell encounters harsh environmental conditions such as hypoxia, malnutrition, or oxidative stress [6,7,8,9]. However, under certain circumstances, like adenoviral infection, autophagy can induce a cell death [10,11,12,13,14]. Some investigators believe that the autophagic type of cell death resembles the process of cellular self-consumption [15,16] as in the course of replication, the adenovirus utilizes the cell’s autophagic machinery, which enhances the infected cell lysis [11]. Generally, at the beginning of the Ad life cycle, autophagic machinery is activated to maintain the infected (host) cell’s viability for successful delivery of the viral DNA and expression of adenoviral structural proteins [12]. Later, the infected cell lysis allows dissemination of the viral progeny to neighboring cells to continue the infection cycle. Thus, just like the ancient Roman dual-headed God of duality, Janus, autophagy controls both the cell death and the cell survival.

Both CRAds and the wild-type adenoviruses are known to trigger autophagy activation [11,17]. Some experimental evidence suggests that Ad directly induces autophagy with the virus-specific proteins E1A and E1B early in its infection cycle via release and activation of the transcription factor E2F1 and upregulation of the Beclin1 expression, respectively [18]. However, since the adenovirus reprograms the host cell’s metabolome to create suitable conditions for its replication, the adenovirus-activated autophagy and the resulting induction of cell death are also expected to be accompanied by metabolic alterations. For example, studies performed by Yacoub and Park et al. demonstrated the accumulation of ceramide/dihydroceramide, elevation of Ca^2+^, production of reactive oxygen species (ROS) (peaking at 12 h post-infection), and the subsequent activation of autophagy in glioma cells 12 h after infection with a recombinant IL-24-armed adenovirus [19]. Ceramide synthesis occurs mostly de novo since a knock-down of ceramide synthase 6 (which regulates C16 dehydro-ceramide levels) results in reduced ceramide levels [19]. However, acidic sphingomyelinase, an enzyme that converts sphingomyelin (a sphingolipid of the plasma membrane) to ceramide [20], also modulates ceramide levels, although to a lesser extent [19]. Inhibition of de novo ceramide synthesis blocks Ca^2+^ elevation, and thus decreases ROS levels (a more detailed mechanism for the impact of calcium levels on ROS formation will be discussed below) [19]. The authors speculate that the elevated ROS levels lead to autophagy activation between 24 and 48 h post-infection, and subsequently to cell death [19,21].

Additional evidence of ceramide’s importance in adenoviral infection comes from the study of Kanj et al., which reported an adenoviral infection-induced de novo synthesis (from serine and palmitoyl-CoA) and accumulation of sphingolipid ceramide in breast carcinoma and lung adenocarcinoma cells that preceded and were prerequisite for the cell lysis in the absence of any evidence of apoptosis [22]. According to the above report, the ceramide levels increased steadily from 24 h post-infection until the late phase of the wild type Ad replication cycle. As suggested by Kanj et al., ceramide accumulation is necessary for the lytic phase of Ad infection, whereas the ceramide pathway is utilized by adenovirus to regulate serine/arginine-rich (SR) proteins during infection, which promotes the viral RNA maturation and thus ensures the onset of the late (lytic) phase of the viral replication. However, the molecular mechanism of this ceramide accumulation remains unclear.

**Figure 1:**
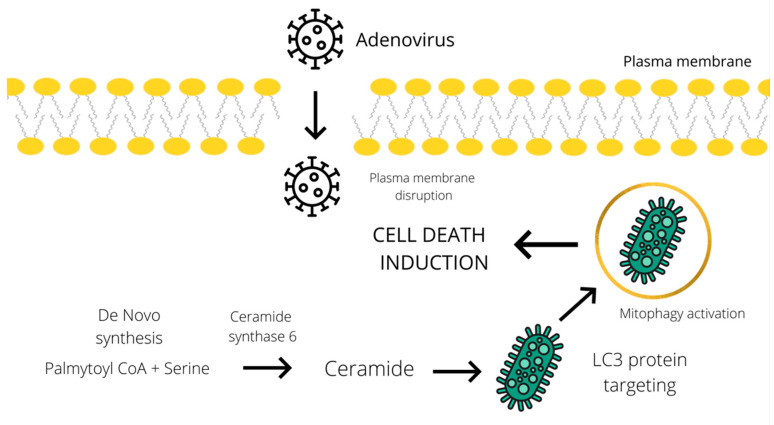
Schematic representation of the tentative cell death mechanism operating through a ceramide-mediated mitophagy. Adenoviral infection induces ceramide accumulation through de novo synthesis from palmitoyl CoA and serine [19]. Subsequently, ceramide mediates the LC3 binding to the mitochondrial membrane and promotes lethal mitophagy leading to autophagic cell death [25].

Furthermore, an increase in ceramide level has been implicated in the activation of the CD95 death receptor, induction of autophagy, and enhancement of cytotoxicity [23,24]. Knock down of ceramide synthase 6 suppressed autophagosome formation and cell death in breast cancer cells. According to the study of Cruickshanks et al., ceramide species activate protein phosphatase 2A (PP2A) by binding to and sequestering its inhibitor. Several studies showed that the accumulation of C18-ceramides on the surface of the mitochondrial membrane leads to their binding to LC3 protein, which could promote a lethal mitophagy in head and neck squamous cell carcinoma [25,26]. Another tentative mechanism of ceramide-mediated autophagy induction, suggested by Edinger et al., involves triggering a bioenergetic crisis by severely limiting cellular access to extracellular nutrients through downregulation of the expression of nutrient transporters [27]. Despite several hypotheses, the actual molecular mechanism of ceramide-mediated cytotoxic autophagy induction has proved difficult to determine. Similarly, oncolytic adenoviruses could induce autophagy and the subsequent cell death by stimulating the accumulation of ceramide species (Figure 1). In addition to all the above, an increase in ceramide levels can be linked to the induction of both apoptosis and necroptosis, which will be described in the subsequent chapter dedicated to these types of cell death.

It is well-known that adenoviral infection is complemented by an increased glucose consumption and lactate secretion by the infected cells [28]. This resembles the frequently observed phenomenon of lactate accumulation in cancer cells under hypoxic conditions due to the anaerobic glycolysis switch, known as the Warburg effect. Furthermore, the accumulated lactate is a substrate for lactate dehydrogenase B, which is thought to increase autophagy in cancer cells [29]. Specifically, the activity of lactate dehydrogenase B contributes to nicotinamide adenine dinucleotide phosphate (NADH) and H^+^ production [29]. According to Brisson et al., hydrogen protons accelerate lysosomal acidification, vesicle maturation, and intracellular proteolysis. Thus, it can be assumed that an oncolytic adenovirus infection may contribute to lactate overaccumulation and the subsequent autophagic cell death induction.

The adenovirus is also capable of mediating changes in the cellular levels of acetyl-CoA. A study by Carinhas et al. confirmed that acetyl-CoA production during adenoviral infection increases two-fold due to the elevated activity of the citrate lyase [28,30]. Possibly, the overproduction of this enzyme is related to the activation of glycolysis and the reduction in the number of acetyl-CoA molecules entering the TCA cycle. The citrate lyase converts citrate into acetyl-CoA and cures the substrate deficiency. Interestingly, an excess of acetyl-CoA is capable of promoting autophagy via inhibition of the acetyltransferase EP300 [31]. Thus, an adenoviral infection could stimulate autophagy indirectly via the induction of acetyl-CoA accumulation.

Infection of prostate cancer cells with an oncolytic adenovirus Ad5/3-D24-GMCSF associated with the autophagy induction and adenosine triphosphate (ATP) release represents yet another example of metabolome shift. Some studies have confirmed that ATP release from dying cells is associated with autophagy [32]. Subsequently, ATP acts as a damage-associated molecular pattern (DAMP) molecule for innate immunity factor recruitment and adaptive immunity activation [33].

Thus, an adenoviral infection may induce autophagy and subsequent cell lysis, either directly or by affecting the cell’s metabolome. Based on all the above, it is plausible to suggest that metabolite accumulation and release could be both the cause and consequence of autophagic cell death.

## 3. Adenovirus Infection-Associated Metabolome in the Regulation of Apoptosis

Apoptosis is a well-studied form of programmed cell death that occurs through caspase activation [34,35]. It results in cell shrinkage, DNA fragmentation, and the formation of apoptotic bodies consumed by macrophages [9,36]. This type of “silent” cell death is not accompanied by inflammation and stays immunologically asymptomatic with minimal damage to surrounding tissues [35,36]. An apoptotic mechanism is triggered by a great variety of stress factors including DNA damage [37], hypoxia [38], and an adenoviral infection [39]. Interestingly, the adenoviral E1A protein is thought to enhance tumor cell’s sensitivity to apoptosis [40,41]. As mentioned previously, an adenoviral infection is accompanied by robust changes in the infected cell’s metabolome. Thus, in theory, the adenovirus is capable of regulating apoptosis via metabolomic shifts.

Numerous studies have reported alterations in acetate metabolism at the time of adenoviral infection or during the accumulation of the virus-specific proteins in the infected cells. For example, by using proton nuclear magnetic resonance (H-NMR) spectroscopy, Silva et al. observed an increase in acetate consumption in the HEK293 culture during adenovirus serotype five (Ad5) infection [28,42]. On the other hand, in another study, Madhu et al. observed an augmented acetate secretion in IMR90 cells transformed with the adenoviral E1A protein accompanied by RAS [28,43]. It is worth mentioning that more than 50 samples were analyzed in this study, so the obtained data are statistically significant and accurate. In the above-mentioned studies apoptosis was not analyzed, but the involvement of acetate in triggering apoptosis has been evidenced by other studies. For instance, Marques et al. reported acetate-induced apoptosis in colorectal carcinoma cells elicited via the lysosomal membrane permeabilization [44,45]. On the other hand, Jan et al. noted that apoptosis in colorectal carcinoma cells could be stimulated by short-chain fatty acids produced by the gut microbiotae [46]. Interestingly, acetate has been shown to prevent apoptosis in gastric mucosa cells. Liu et al. noted that acetate alleviates ethanol-induced oxidative stress and down-regulates the Bcl-2-associated X protein (BAX) and caspase 3 [47]. Perhaps, the proapoptotic effect of acetate takes place exclusively in tumor cells, whereas in normal cells it tends to have a protective effect. Thus, adenoviral infection is capable of triggering apoptosis indirectly via affecting the acetate metabolism and influencing both acetate secretion and consumption selectively in cancer cells.

Another example of an adenovirus-mediated metabolomic shift, as already mentioned above, is the elevated level of ceramides [22]. Ceramides have been suggested to play an important role in the apoptotic pathways. For instance, Cheng and colleagues revealed that the C6 ceramide pathway promotes apoptosis in multiple myeloma cells by enhancing caspase 3/9 activity [48]. Furthermore, ceramides were found to trigger an intrinsic apoptotic pathway via initiating mitochondrial outer membrane permeabilization (MOMP) [49]. MOMP occurs through the formation of ceramide channels, which are large enough to allow protein translocation [49]. The channel formation can be inhibited by Bcl-xL [49]. Besides, ceramides have been shown to enhance drug-induced apoptosis in hepatocellular carcinoma by induction of ROS and mitochondrial depolarization, which promote a caspase-dependent apoptosis accompanied by caspase 3/9, Bax, Bcl-2, and cytochrome c expression [50]. Therefore, an adenoviral infection could be the reason for apoptosis induction through ceramide-mediated mitochondrial alterations.

As stated previously, the viral life cycle in the cell requires conditions capable of supporting its replication cycle. To achieve those, adenoviral infection stimulates an increase in glucose and lactate uptake [42,43,51], in addition to other effects on the cell’s metabolism. These metabolic alterations have deep consequences on the cell’s physiology. For example, Kasinskas et al. reported induction of apoptosis in tumor tissue culture as a result of rapid glucose and lactate uptake [52]. The authors hypothesized that the rapid nutrient uptake-induced depletion of carbon sources induces apoptosis. Thus, it appears that an adenoviral infection dramatically reprograms cell metabolism to provide itself a suitable environment in the host cells with an amenable amount of energy and substrates, however, the cells, unable to sustain this dramatic change in metabolism, launch an apoptosis program. Adenoviral infection is typically associated with the initiation of a cell death mechanisms in the host cells. However, some adenovirus-induced metabolites are known to prevent cell death, particularly apoptosis. For example, two studies conducted by Carinhas et al. and Thai et al. have demonstrated an increase in asparagine production and its intracellular accumulation in the course of adenoviral infection [53,54]. In this regard, Zhang et al. came up with the idea that asparagine acts as a suppressor of apoptosis in tumor cells during glutamine depletion, by entering the tricarboxylic acid (TCA) or the Krebs cycle [55]. This offers an attractive strategy for constructing a genetically-modified adenoviral genome encoding the asparaginase transgene to induce apoptosis in infected tumor tissue as a way of augmenting the vector’s oncolytic efficacy. Thus, adenovirus-induced metabolites are capable of triggering apoptosis through global metabolome alterations. A more thorough investigation of metabolites involved in both adenovirus-mediated metabolome changes and cell death triggering mechanisms may help in discovering new molecular targets to facilitate oncolytic virotherapy.

## 4. Adenovirus Controls Necrosis and Necroptosis with the Help of Cellular Metabolites

Unlike the cell death mechanisms mentioned above, necrosis represents a non-programmed, energy-independent form of cell death that is characterized by adenosine triphosphate (ATP) depletion, influx of calcium, intracellular organelle swelling, nuclear degradation, loss of plasma membrane integrity and spilling of the cell content into surrounding tissues [34,35]. Necrosis is thought to be an “accidental” cell death caused by external factors such as physical damage, lack of blood supply (hypoxia), or inflammation [34]. However, the necrotic process can sometimes be triggered by a cell’s internal program. This mechanism is called necroptosis, a programmed necrotic cell death controlled by mixed lineage kinase domain-like protein (MLKL) and receptor-interacting proteins 1 (RIPK1) and 3 (RIPK3) [9,35,56].

Numerous studies have reported that adenovirus is capable of inducing both necrosis and necroptosis [57,58,59,60,61,62]. However, it is worth mentioning that necrosis and necroptosis are morphologically indistinguishable [63], thus, they cannot be identified simply by a histological examination. Instead, the enzymatic activity should be measured to precisely determine the cell death type. Essentially, it remains unclear if adenoviral infection induces necrosis or necroptosis. In our opinion, “necroptosis” is a more appropriate mechanism in the context of adenovirus-mediated cell death because necrosis typically occurs as a consequence of external injury, while necroptosis is the result of an intracellular program launched by internal changes like adenoviral infection or some metabolic fluctuations promoted by it.

Despite the growing number of studies in the field, the mechanism of necroptosis remains poorly understood. According to some data, the production of ROS is essential for necroptosis to occur and is required for peroxidation of membrane structures and subsequent induction of oxidative burst resulting in disruption of cellular structures [63,64]. Some studies have demonstrated that ROS are produced by the mitochondrial complex I [63]. In 2012, Huang et al. reported the induction of necroptosis and a concomitant significant increase in ROS production in a hepatoma cell line upon its infection with an interferon-β-armed oncolytic adenoviral vector [58]. This study’s results are consistent with the possible role of adenovirus infection in ROS production. Moreover, a dramatic decrease in the intracellular levels of ATP was noted, which is in agreement with the occurrence of ROS-mediated necroptosis as an energy-independent process. The observed ATP deficiency was probably related to the stimulation of DNA synthesis induced by adenoviral replication. Alternatively, the reduction in ATP levels could be a consequence of necroptosis induction by the Receptor-interacting protein-1 (RIP1)-dependent inhibition of adenine nucleotide translocase (ANT) [65] that transports Adenosine Di Phosphate (ADP) into mitochondria. Interestingly, the intracellular ATP levels were recovered 48 h post-infection and indicated a switch from necroptosis to apoptosis, as the intracellular ATP levels determine the mechanism of cell death [58,66].

ATP level fluctuations were also observed as a result of infection with an oncolytic vector, Enadenotucirev or wild-type adenovirus serotypes 3, 5, and 11 [67]. Interestingly, Dyer et al. noted a temporary increase in ATP levels in the beginning of infection due to the adenovirus-mediated boost in cell metabolism [67,68]. However, subsequently, the elevated ATP levels dropped dramatically, which might be a consequence of the enhanced DNA synthesis during the viral replication, as already mentioned above. Moreover, an extracellular ATP release has also been observed during necroptosis, which indicates the loss of the plasma membrane integrity [67]. No signs of apoptosis or necroptosis were observed in this study and no evidence for the activation of caspases or RIP1 was found either. Based on these data, the authors suggested that the observed cell death mechanism resembled oncosis, an ischemic type of cell death caused by ATP depletion and characterized by cell swelling and the formation of large single-cell blisters, which is thought to reflect the loss of control of cellular ion gradients. On the other hand, intracellular calcium levels increased at the time of infection in all the viruses. Intracellular calcium is important for the adenoviral structural protein fiber secretion mechanism [69]. Perhaps, it is related to the development of acidosis due to the lactic acid accumulation caused by the adenovirus-mediated stimulation of glycolysis [28]. While intracellular pH decreases, acid-sensing ion channels become activated and induce calcium influx [70,71]. Another possible mechanism of intracellular calcium accumulation is calcium flow through tiny pores in the membrane and lysosomal exocytosis induced by adenoviral E4 protein [20]. However, calcium accumulation could also be promoted simply by ATP depletion and subsequent calcium pump inactivation. Calcium itself is capable of stimulating oxidative phosphorylation in mitochondria and the subsequent enhancement of the mitochondrial complex I activity, which results in ROS production [72]. Moreover, an increase in intracellular calcium could trigger a change in mitochondrial permeability [56,73]. Thus, the adenovirus-induced increase in matrix calcium levels could theoretically trigger a necrosis-like cell death mechanism. For example, another study involving Enadenotucirev and adenovirus serotypes 11 and 5 demonstrated augmented oncolytic activity caused by a drug-mediated increase in the calcium influx [74]. A similar mechanism exists for mitochondrial- and necrosis-dependent heart failure, where a necrotic cell loss is associated with calcium overload [75].

**Figure 2:**
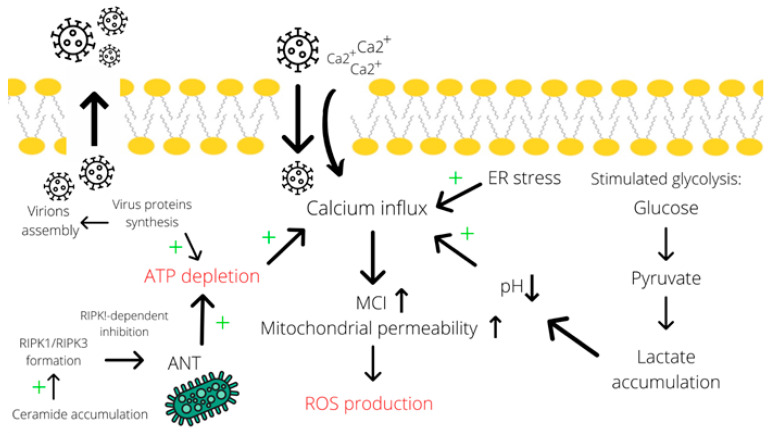
Calcium influx contributes to the induction of necrotic cell death. Calcium influx is promoted by many factors, including endoplasmic reticulum stress, acidosis (due to lactate accumulation) [70,71], and ATP depletion (particularly due to assembly of virions and RIPK1-dependent inhibition of ANT in the mitochondrial membrane mediated by ceramide accumulation). Subsequently, an elevated concentration of calcium induces ROS production through increasing mitochondrial permeability [56,72,73]. A simultaneous decrease in ATP levels and the induced ROS production result in necrotic cell death. Abbreviations: ER—endoplasmic reticulum, ANT—adenosine nucleotide translocase, MCI—mitochondrial complex I, LDH—lactate dehydrogenase, “+”—increase effect.

As discussed previously, some evidence supports the accumulation of ceramide species in the cell upon adenoviral infection [22]. Numerous studies implicate ceramides in the initiation of cell death, particularly, by the necroptosis mechanism. For instance, Thon and colleagues described a TNF-promoted caspase-independent type of cell death that occurs through ceramide accumulation [76]. Besides, the authors reported that the absence of RIP1 prevents ceramide accumulation and the subsequent cell death. Furthermore, Zhu et al. characterized C2-ceramides as second messengers and proved their involvement in the caspase-3-independent apoptosis and necroptosis in HNSCC cells [77]. Even though the mechanism of cell death induction by ceramides is still poorly understood, the contribution of these metabolites to the necroptosis process is quite evident. Therefore, by contributing to ceramide accumulation in the cell, adenoviral infection is also capable of inducing necroptosis (Figure 2).

The necrotic cell death-associated changes in the metabolome are complemented by leakage of the cell content into the surrounding tissue. Subsequently, the released DAMP molecules, including ATP, calreticulin, or high mobility group box 1 protein (HMGB1), act as immunostimulators leading to the development of inflammation [61], which may potentially lead to the tumor-specific immunity activation [61]. Viral infection-induced immune response activation may help the dormant immune system to recognize the tumor. Thus, the construction of adenoviral vectors, capable of provoking a necrotic cell death by inducing ATP depletion and ROS production, may potentially help in eliciting anti-tumor responses and enhance the vector’s oncolytic efficacy.

## 5. Conclusions and Future Directions

**Figure 3:**
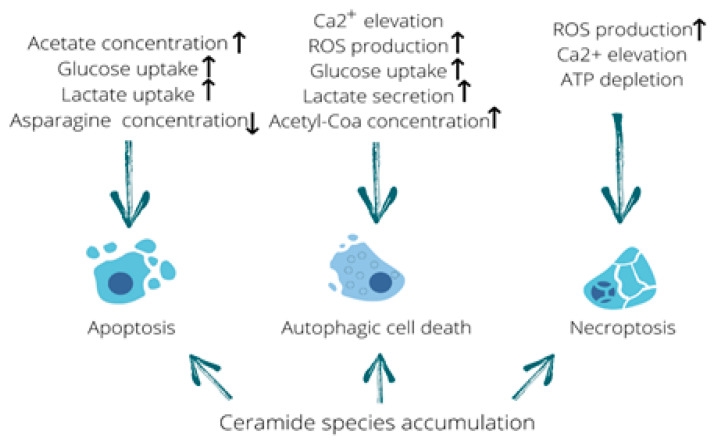
Metabolome fluctuations that trigger different types of cell death. Elevated acetate levels stimulating uptake of glucose and lactate along with asparagine deficiency can be linked to the induction of apoptosis. Calcium influx, production of reactive oxygen species (ROS), increased glucose uptake and lactate secretion as well as an elevation in acetyl-CoA concentration are all capable of triggering autophagic cell death. In addition, ROS production, calcium elevation and ATP depletion are known to induce necroptosis. Furthermore, accumulation of ceramide species is capable of triggering all three types of cell death described in this review. Up arrow means it increases and down arrow means it decreases.

This review highlights the ability of human adenovirus to cause alterations in the infected cell metabolome since viral invasion, viral protein synthesis, and the assembly of the progeny virions occur through the utilization of cellular resources and the induction of numerous metabolic pathways in the host cell. In some cases, metabolomic shifts lead to the induction of cell death because altered metabolites modify the activity of enzymes and cooperate with cell organelles promoting their structural disruption and the loss of functionality. As outlined in this review, in most cases, alterations in metabolite concentrations represent the cause of cell death and not its consequence. Shifts in metabolomic profiles may lead to the induction of various types of cell death such as apoptosis, necroptosis, autophagy or both apoptosis and autophagy (Table 1) [10,13,14,78,79,80,81,82,83,84]. Furthermore, incorporation of a transgene into the adenoviral genome can enhance apoptosis in Ad-infected cells both in vitro and in vivo [85,86,87]. Each type of cell death mentioned above is accompanied by a specific pattern of metabolomic alterations, which the cell is unable to sustain. As illustrated above, ceramide species promote all three types of cell death. Interestingly, while apoptosis stays immunologically invisible, autophagy and necroptosis are complemented by the release of DAMP molecules that may lead to tumor-specific immunity activation. Consequently, the adenovirus-mediated induction of oncolysis, accompanied by immune stimulation, promotes more effective tumor eradication (Table 1). To summarize the highlights of this review, all the described metabolic changes that trigger different types of cell death have been outlined in a table format (see Figure 3).

While researching the literature for this study, we noticed that with regard to oncolytic adenoviral infection, researchers give higher priority to the analysis of cellular proteomics relative to that of metabolomics. In our opinion, a more thorough investigation of alterations in the metabolic profiles of Ad-infected cells could greatly facilitate our understanding of the precise mechanisms of adenovirus-mediated metabolism reprogramming and cell death induction. Eventually, the genetic modification of already existing oncolytic vectors, based on the advanced knowledge in the field of cell metabolomics, could help in improving the oncolytic efficacy of those vectors.

Box 1 **Metabolite Analysis: The Adenovirus-Mediated Reprogramming of Cell Metabolism.**

Metabolite analysis is a rapidly-evolving approach that involves the characterization and comparison of certain cell’s metabolic profiles. Metabolites are low molecular weight organic compounds, generated in the cell as intermediates or final products of metabolic pathways. Many factors, including adenoviral infection, can cause dramatic metabolic changes, which are reflected by alterations in metabolic profiles. Adenoviruses are small, non-enveloped, double-stranded DNA viruses that transfer their genetic material into infected (host) cells following a receptor-mediated attachment to the cell’s plasma membrane and internalization of the attached viral particles. The viral DNA is then translocated to the nucleus, where its transcription occurs; subsequently, virus-specific mRNAs accumulate and undergo translation in the cells cytoplasm to produce viral proteins. Finally, the viral proteins and the replicated viral DNA genomes are assembled into infectious progeny virions, which are released from the host cell, causing its lysis. During its life cycle in the infected cell the virus utilizes cellular resources such as ATP, amino acids, etc., to support its replication. As a result, the cell suffers metabolite deficiency resulting in disruption of the TCA (Krebs) cycle and the loss of energy. Interestingly, although adenoviruses are non-enveloped, they still upregulate the cell’s lipid metabolism [88]. Moreover, adenovirus stimulates glycolysis, possibly because it is a fast way of generating vital energy. Some data suggest that the viral proteins activate glycolysis to convert glucose into nucleotides [89]. Besides, the products of the viral life cycle interact with intracellular enzymes, altering their activity.

As we have illustrated here, adenoviruses dramatically reprogram cell metabolism and induce metabolomic shifts. Eventually, such changes in metabolism can lead to various consequences for the metcell, including the induction of cell death.

Box 2 **Cell Death Induction with the Help of Metabolites.**

Metabolites are low molecular weight organic compounds generated in the cell as intermediates or final products of metabolic pathways. Shifts in metabolic profiles may induce disruption of vital cellular functions and promote cell death activation. For instance, as a result of ATP depletion, all energy-dependent processes are hampered, including the ion channel pump activity. Besides, the depletion of amino acids also leads to a hypo-energetic condition due to inhibition of the TCA cycle, since amino acids ameliorate the deficiency in the TCA cycle metabolites. Consequently, ATP deficiency and the lack of energy result in cell death induction as the cell is unable to maintain its homeostasis. Cell death can result not only from a deficiency of metabolites, but also from their overaccumulation. For example, an intracellular increase in ceramides, which frequently reflects disruption to the plasma membrane, provokes the induction of different types of cell death through various molecular mechanisms, commonly affecting mitochondria. In most instances, the effects of metabolites on cell viability are exerted by specific molecular mechanisms, all of which significantly alter the cell’s homeostasis. Some authors have even proposed the existence of specific metabolic checkpoints that control the cell fate. Thus, the dramatic metabolic shifts caused by various endogenous and exogenous factors are capable of ultimately promoting cell death.

## Figures and Tables

**Table 1:** The three types of adenovirus-induced cell death.

Cell Death Type	Autophagic Cell Death	Apoptosis	Necrotic Cell Death
General description	Catabolic process that presents degradation of intracellular components in autophagosomes.	Form of programmed cell death that proceeded through caspase activation [32,33]	Necrosis: “accidental” cell death caused be excessive damage. Necroptosis: programmed necrotic cell death, controlled by MLKL and RIPK1/3
Role	Pro-survival role to maintain cell’s homeostasis under harsh conditions [6,7,8,9]. While coping with excessive stress switches to mechanism inducing cell death resembling self-consumption [10,11,12,13,14]. Can be triggered when apoptosis is inhibited.	Elimination of damaged or infected cells without immune response activation	Controlled and uncontrolled death mechanism in the context of heavy damage. Necroptosis can be triggered when apoptosis is inhibited.
Morphology	Formation of double-membraned autophagosomes surrounding cell’s organelles.	Cell shrinkage, DNA fragmentation and formation of apoptotic bodies [9,34].	Cell swelling, nuclear degradation, plasma membrane destruction and cell contents’ spilling into surrounding [32,33]
Markers	p62, LC3B expression	Caspase activation, DNA fragmentation	MLKL, RIPK1/3 expression
Immune response	Activated due to DAMP release	Immunologically asymptomatic	Activated due to DAMP release
Metabolic triggers	Ceramide accumulation, increased glucose consumption and lactate secretion, acetyl-CoA accumulation. Accompanied by ATP release.	Ceramide accumulation, acetate accumulation, rapid glucose uptake, glutamine and asparagine depletion	Ceramide accumulation, ATP depletion and ROS production

## Data Availability

All data are contained within the article.
